# A Lecture on the Physiology of Digestion

**Published:** 1846-06

**Authors:** 


### PART III.—BIBLIOGRAPHICAL NOTICES.

ARTICLE VII.

*A Lecture on the Physiology of Digestion*, introductory to a Course of Lectures on the Institutes of Medicine and Materia Medica. Delivered before the Medical Class of the City of New York, at the Session of 1844-5. By MARTYN PAINE, A. M., M. D., Prof. &c. Fourth edition. New York. J. H. Jennings. 1844-5. pp. 24.

The Introductory Lecture, of which the above is the title, though published more than a year since, has just made its appearance upon our table. We notice it, at this time, for the purpose of giving our readers an example, showing the general character of the author's productions.

The Professor begins his remarks by assuming that there are, at this time, in our profession, three distinct classes of medical philosophers, the chemists, vitalists, and chemico-physiologists. The first "of these sects," to use the writer's language, "virtually regards organic nature as a part only of inorganic, endowed with the same properties, and governed by the same laws. It maintains, in short, that there is no real difference between a man and a stone. At the head of this school stands Liebig, the distinguished and able chemist. It is a great and powerful school, but is falling, daily, beneath the weight of its own vast errors and corruptions." "Contrasted with this," continues the Professor, "is the school of vitalism; and to the interests of this school, I may now say, the main efforts of my life have been devoted." * * "Finally, the third school, or that of chemico-physiology, endeavors to form, as it were, a bond of union between the schools of pure vitalism and pure chemistry, though such an alliance be as unnatural as human brains in a block of granite." Dr. Prout is honored with the rank of leader in this last named school. Thus it will be seen that Prof. Liebig and Dr. Prout are ranked with Prof. Paine himself, each as leader and expounder of the views of his own particular school.

The Professor then goes on to make some remarks on the physiology of digestion, in which, without facts or arguments in favor of his own views upon the subject, he makes numerous quotations from Liebig, taken in detached passages, and from the works of this author, in such connection as to make them appear, in his opinion, contradictory and absurd; and concludes by saying, "Such, then, are examples of the medley of contradictions in great fundamental principles, with which the writings of this very extraordinary and successful pretender in medicine, abound."

The writer does not stop with Liebig, but continues in succeeding remarks to speak in the most disrespectful, not to say abusive terms, of such men as Prout, Roget, Carpenter, Thompson and others, equally distinguished, and respected by the medical world for their scientific labors and acquirements.

Whether such harsh expressions towards devoted votaries to the science of medicine, even if their numerous conclusions do not always harmonize perfectly, add either honor or dignity to the name of one, who, to use his own expression, "stands up *alone* in the broad expanse of America in an open defence of the honor and dignity of the profession," we will leave for our readers to determine. W. B. H.

